# Work-Life Conflict Among Higher Education Institution Workers' During COVID-19: A Demands-Resources Approach

**DOI:** 10.3389/fsoc.2022.856613

**Published:** 2022-03-23

**Authors:** Carolina Garraio, Jorge Peixoto Freitas, Sara Isabel Magalhães, Marisa Matias

**Affiliations:** ^1^Department of Psychology - Faculty of Psychology and Education Sciences, University of Porto, Porto, Portugal; ^2^Rectory of the University of Porto, Porto, Portugal; ^3^Faculty of Psychology and Educational Sciences of the University of Porto, Porto, Portugal; ^4^Center for Psychology at University of Porto, Porto, Portugal

**Keywords:** work-life conflict, role overload, work dedication, family-supportive organization perceptions, gender, COVID-19, higher education institution

## Abstract

Higher Education Institutions' (HEI) workers were highly impacted by the COVID-19 pandemic, which magnified gender differences in terms of management of work and personal life. Most studies published so far have primarily focused on a group of HEI workers' (i.e., teachers and researchers), but not on staff members, despite their crucial role for HEI functioning. Following the Job Demands-Resources theory, we aimed to: (i) characterize work-life conflict (WLC) among men and women workers from an HEI (staff and teachers/researchers) during the COVID-19 pandemic; and (ii) explore the major predictors of WLC for both staff and teachers/researchers. This study includes a sample of 262 workers from one Portuguese HEI (*n* = 128 staff members; *n* = 134 teachers/researchers) who answered an online survey. An Independent Samples *T*-Test showed that the reported current WLC was significantly higher for teachers/researchers compared to staff. Moreover, women teachers/researchers showed higher WLC than men. Additionally, using a Repeated Measures ANOVA, we found that the increase in the reported levels of WLC (before the pandemic and currently) was significantly more prominent among teachers/researchers than in the staff group. Regarding the predictors of WLC for both groups separately, a Multiple Hierarchical Linear Regression showed that role overload, conceptualized as a demand, was a predictor for both staff and teachers/researchers. As for potential resources, work dedication negatively predicted WLC for staff, whereas family-friendly organization perceptions predicted less WLC for teachers/researchers. These results highlight the importance of understanding HEIs holistically, by considering workers' individual characteristics such as gender, but also distinct careers inside the institutions. As most European HEIs are currently making active efforts to promote gender-equal academic workplaces, these findings may help them design tailored and effective measures to address employees' work-life balance issues, not only considering gender, but also the different types of demands associated with each group of workers within HEIs.

## Introduction

Higher Education Institutions (HEI) workers' were highly impacted by the COVID-19 pandemic, following the exceptionally new context with abrupt changes associated to social distancing, remote work, and remote teaching (Collins et al., 2020; Sinclair et al., 2020; Meyer et al., 2021). The already existing gender differences regarding work management and work and personal life articulation were magnified (Minello et al., 2021). However, the major demands HEI workers' have faced, along with the most valuable resources, is yet to be addressed. Moreover, most studies published so far have primarily focused on teachers and researchers, but not on staff members (with only a few exceptions, e.g., Charoensukmongkol and Phungsoonthorn, 2021). This group of workers is not only part of the same institutional context but is also a crucial group for supporting and monitoring academic activities. The only study specifically addressing a HEI staff has highlighted the role that supervisor support played in reducing perceived uncertainties of employees whose workplace climate was low in intransigence (i.e., that was not resistant to change) (Charoensukmongkol and Phungsoonthorn, 2021). On the other hand, studies addressing teachers and researchers, portrait a clear picture of the impact that COVID-19 had on daily activities and the possible psychological effects these workers were faced with. More specifically, high expectations for scholarly productivity and the shift to remote teaching represented additional challenges (Mishra et al., 2020) that were intertwined with other individual stressors related to the lockdowns (Hamouche, 2020), which created a vulnerable context for conflicting demands arising from work and family domains (Sinclair et al., 2020).

### Theoretical Background

Research published thus far unequivocally shows that inequalities between men and women have been exacerbated by the pandemic (Minello et al., 2021). For instance, the closure of childcare facilities and restriction of social contacts, deemed as important resources for women with children, intensified the unequal distribution of duties between men and women (Meyer et al., 2021). Working mothers were indeed impacted to a higher extent than men in terms of psychological wellbeing, experiences of negative emotions, and redefinition of family dynamics (Clark et al., 2021), and this was most probably due to the loss of highly relevant resources (Meyer et al., 2021). Among dual-earner parents, mothers have reduced their working time significantly more than fathers to meet their children's caring and homeschooling demands (Collins et al., 2020), while they were also the ones responsible for “emotional labor” as they were trying to keep their relatives calm and safe (Hjálmsdóttir and Bjarnadóttir, 2020). Thus, the COVID-19 and its associated lockdowns created a prone context for the increase of role demands, both from family and work (Sinclair et al., 2020), whose combination may have risen work-family conflict. The work-home resources model (Ten Brummelhuis and Bakker, 2012) states that work-home conflict occurs when contextual demands (e.g., working overtime, many household chores, urgent care tasks) drain personal resources (health, mental resilience, fulfillment). The relationship between these demands and resources is influenced by macro resources (i.e., the economic, social and cultural context in which the person is embedded) which, in this context, have been highly impacted by the pandemic. Therefore, a positive relationship between contextual demands and personal resources has likely been fostered, leading to work-life conflict.

As gendered organizations (Acker, 1990), HEIs tend to reproduce these structural inequalities in practical work activities, and the pandemic context has only made them clearer. Among teachers and researchers, we know that high expectations for scholarly productivity and the shift to remote online learning platforms represented additional challenges (Mishra et al., 2020) which were intertwined with other individual stressors related to the lockdown (Hamouche, 2020). Studies conducted during the pandemic have indeed highlighted gender differences among academics in the way they managed work and personal life tasks. Women researchers and those with young children reported that their ability to dedicate time to their research has been greatly impacted (Myers et al., 2020), along with their ability to submit articles (Staniscuaski et al., 2021). The pandemic has also forced the abrupt introduction of digital modes of course delivery, whose development, preparation and instruction were much time and labor consuming (Mishra et al., 2020). The traditional gendered distribution of household labor has affected men's and women's academic functions, particularly of women academics with children (Staniscuaski et al., 2021; Yildirim and Eslen-Ziya, 2021). For instance, men perceived their work as not flexible enough to meet other obligations, such as their children's homeschooling. Contrarily, despite similar amounts of obligations, women perceived their work to be flexible enough to conciliate with other tasks, such as household related duties and children homeschooling (Górska et al., 2021). On the other hand, these differences may also stem from inequalities that are internal to academia, where women are seen as most responsible for material and emotional caring for their colleagues and students (Minello et al., 2021; Pereira, 2021). Overall, while men seem to have segmented their work-non-work boundaries, women appear to have blurred frontiers between work tasks and household responsibilities, which created a vulnerable context for conflicting demands from work and family (Sinclair et al., 2020). It is worth noticing that work-life conflict is a predictor of wellbeing variables, such as burnout, general wellbeing and subjective physiological health problems (Steffgen et al., 2020).

Given the different nature of tasks of teachers/researchers and staff, the way each of these experienced the impact of the pandemic may have also been different. However, while there is a great number of studies that focus on academics' experiences during the pandemic, for staff members they are scarce. Staff members are a heterogeneous group of workers (i.e., from students' registrations offices, IT support, accounting and finance, research and innovation, among others). Nevertheless, all these workers and functions are crucial to the functioning of a HEI and all of them had their work setting changed due to the pandemic (e.g., starting remote work). It is therefore expected that changes to work and family boundary management, to household distribution and to work and family reconciliation also occurred to staff members.

Following the Job Demands-Resources theory (Bakker and Demerouti, 2007; Bakker and de Vries, 2021), we aimed to understand what were the specific demands and resources that both staff and teachers/researchers have been faced with in the pandemic context. Job demands are aspects of work that require high personal efforts and therefore associate with physical and psychological costs (Bakker and Demerouti, 2007). Complementary approaches to this model distinguish challenging job demands, that may play a motivational role, and hindrance job demands, where the latest represents undesirable or excessive constraints that interfere with the individual's ability to achieve valued goals (LePine et al., 2005). Among these hindrance job demands, we highlight role overload, which is the perception that demands from a person's role (e.g., from work or family) are higher than the person's energy or time resources to fulfill their requirements (Duxbury et al., 2008). Role overload seems to capture the complexity of experiences that workers faced during the pandemic, considering individual, work and family demands that may lead to a feeling of overload. On the other hand, job resources are the physical, psychological, social or organizational aspects that are necessary not only to deal with job demands and their costs, but also to foster personal growth, learning and development (Bakker and Demerouti, 2007). Job resources, such as social support may thus buffer negative impacts of work demands. When job demands are higher, stable resources, both personal and organizational, are key to reduce or prevent job strain (Bakker and de Vries, 2021). Indeed, considering the great impact that the pandemic has posed on individuals' family lives, family-supportive organization perceptions may have played a key role in promoting a healthy management between work and personal life tasks. These perceptions represent the worker's belief that their organization is supportive of their family role, namely through the availability of policies and benefits that help employees manage their work and family lives (Booth and Matthews, 2012). Additionally, job resources may also instigate motivational processes (Bakker and Demerouti, 2017). Thus, work dedication, the feeling that one's work is meaningful, a source of inspiration and pride (Schaufeli et al., 2006), may have been an important dimension to consider while working in a challenging time as the COVID-19 pandemic.

Within an HEI environment, a recent study applied a job demands and resources framework to identify which were the ones most experienced by academics (Naidoo-Chetty and Du Plessis, 2021). Resources were grouped into organizational (social support) and personal (meaningful work) resources, while demands fell into three categories: quantitative (e.g., publication pressure, overburden with workload, competing time demands), qualitative (e.g., work/home balance), and organizational (e.g., lack of structural resources) (Schaufeli, 2017; Naidoo-Chetty and Du Plessis, 2021). The authors noted that there were more quantitative than qualitative demands identified by academics, which could indicate that they are dealing with great amounts of work, pointing to role overload as an important dimension to be considered. Even though this data did not derive from the pandemic context, it is still very informative as to the extent to which demands and resources may have been most relevant during COVID-19 for each working group (i.e., teachers/researchers, and staff members) among HEIs.

### The Current Study

A pre-pandemic study (Duxbury and Halinski, 2014) has already stated that telework may be helpful to meet high work demands, but the same does not happen regarding high family demands. The authors have suggested that when family demands at home are higher, individuals tend to segment their roles, which may explain why remote work may not be as beneficial in a situation where non-work demands are higher, which was most likely during the pandemic. Additionally, even before the pandemic, academics were already concerned with the incorporation of technologies in higher education directed to blended learning, and were finding it difficult to adjust, mainly due to a feeling that they were not being cared for by their university (Huang et al., 2021). We therefore expect that the group of staff and of teachers/researchers have perceived an increase on the levels of WLC, when comparing with the pre-pandemic period (H1).

Thus, the sudden shift to remote work imposed by the COVID-19 pandemic to workers from HEIs, instead of helping reduce work demands, may have had negative consequences as far as non-work demands are concerned. In this study, by taking stock on the evidence gathered thus far about the heightened gender inequalities due to remote work and lockdowns related to COVID-19 pandemic, we expect that the levels of current WLC are perceived as higher among women teachers/researchers and staff than in men (H2).

We further want to explore how the two major group of workers within HEI perceived their levels of WLC, and therefore pose the following research question: Do staff and teachers/researchers experience different levels of WLC during the pandemic? (RQ1).

Also, considering a Job Demands-Resources approach, and applying it to the experience of HEI workers', we aim to explore how job demands, addressed by the perception of role overload (quantitative demand), and job resources, addressed by family-supportive organization perceptions (organizational resource) and work dedication (personal resource) (Schaufeli, 2017), may act as predictors of WLC for staff and teachers/researchers. As the pandemic has exacerbated gender differences in terms of work-family reconciliation, being a woman and having dependent children are also considered as additional demands in the prediction of WLC. Since studies on these two groups of workers are rather scarce, we again pose a research question to address this issue: What are the main predictors of current WLC for staff and teachers/researchers during the pandemic? (RQ2).

Ultimately, we aim to provide a comprehensive view of the overall functioning of a HEI, accounting for the experiences of men and women teachers/researchers and staff members.

## Methods

### Participants and Procedure

This study was approved by the Ethics Committee of the authors' institution (112/CEUP/2021). Data was collected between June and September 2021 through an online survey that was hosted at the university surveys platform. Participants were recruited via email and two reminders were sent by the Communications Office to all University workers (researchers, teachers, and staff members).

The final sample included 262 participants from one Portuguese public university, 128 staff members (48.9%; *n* = 80 women; *n* = 28 men) and 134 teachers/researchers (51.1%; *n* = 69 women; *n* = 34 men), aged between 25 and 69 (*M* = 47.22; *SD* = 10.72). Close to half of staff are either married (30.5%) or have a partner (17.2%), and the same happens for teachers/researchers (38.5% are married; 15.7% have a partner). Among staff, 46.9% have children, while 58.2% of teachers/researchers do. As for the type of contract held with the institution, 57% of staff have an open-ended/permanent contract, while this percentage decreases to 49.3 among teachers/researchers. When analyzing the association of these variables with the group of workers, we found an association between being a teacher/researcher or staff with having children, marital status and type of contract. See Table 1 for more details about the sample.

**Table 1:** Sociodemographic and work characteristics of teachers/researchers and staff.

	**Teachers/Researchers (*n* = 134)**			**Staff (*n* = 128)**		
	***%***	***M***	***DP***	***%***	***M***	***DP***
**Gender**						
Men	25.4	*-*	*-*	21.9		
Women	51.5	*-*	*-*	62.5		
Age		49.9	11.1		44.6	9.7
Having children	**58.2**			46.9		
Taking care of children daily	26.1			23.4		
Taking care of dependent adults daily	16.4			14.8		
**Marital status**						
Single	7.5			**19.5**		
Having a partner	15.7			17.2		
Civil Union	5.2			2.3		
Married	38.1			30.5		
Remarried	1.5			1.6		
Divorced/separated	9.0			13.3		
Widow	1.5			0.0		
**Employment status**						
Full professor/coordinator researcher|Management 3^rd^ degree	8.2			0.0		
Associate professors/Principal researchers|Management 2^nd^ degree	23.9			7.9		
Assistant Professor/Assistant Researcher|Management 1^st^ degree	44.0			64.8		
Lecturer/Junior Researcher/Researcher|Operational Assistant	23.1			21.1		
Technical Assistant	-			2.3		
Graduated Staff	-			0.8		
IT	-			0.8		
**Type of contract**						
Open-ended/permanent	49.3			57.0		
Permanent tenured	16.4			**28.1**		
Fixed-term	**15.7**			3.9		
Uncertain term	12.7			10.2		
Part-time	3.0			0.0		
Hourly paid	1.5			0.0		
Zero hours	0.7			0.0		
Contract for service	0.0			0.0		
Third party funded fellowship	3.0			0.0		
University funded fellowship	0.7			0.0		

*The n varies between 213 and 262. Given the existence of missing values, the sum of percentages in gender and marital status does not equal 100*.

*The **bold** values indicate that the adjusted residuals are >1.96 and the **bold underlined** indicate that they are >-1.96, i.e., there are more (fewer) cases in this cell than expected if the variables were independent*.

For staff members, the response rate was 7.7%, while for teachers/researchers, it was of 4.3%. In terms of representation of the HEI under study, the sample was able to capture an approximate picture of its population, where 36.7% are staff members, of which 69.9% are women, and 65.3% are teachers and/or researchers, of which around 51.7% are women. Additionally, out of the 18 organic units (faculties and autonomous services) of the institution, we got replies from workers from 17 of them. The organic unit from which we did not receive any answer is also the one with the smallest number of workers in the institution−18 in total. Our sample size encompasses a margin of error of 5.9%.

Considering the possible negative impacts of forced answering in data quality of online surveys (e.g., dropout rates and faking behavior), the answers in this survey were not mandatory (Sischka et al., 2020). For this reason, some questions were not replied by the whole sample. However, we did not exclude these participants and instead treated these values as missing.

### Measures

#### Sociodemographic and Work Characteristics

Participants indicated their gender, age, marital status, existence of dependent children, along with work characteristics such as their type of contract, employment status and number of hours of work per day.

#### Work-Life Conflict

Work-life conflict was assessed with a scale retrieved from the European Social Survey (2011), specifically from the module of Work, Family and WellBeing. This scale is composed of 6 items (e.g., How often do you feel too tired after work to enjoy the things you would like to do at home?), rated on a 5-point Likert scale. This scale was presented twice in our survey, firstly referring to before 2020 (before the pandemic), and then to the current moment when data was being collected (during the pandemic). Each varied between (1) *Never* and (5) *Very frequently*. Both scales have a good reliability (Before 2020 - ω = 0.828, 95% CI [0.80, 0.86]; Current moment - ω = 0.820, 95% CI [0.80, 0.87]).

#### Work Dedication

Work dedication was assessed with the Dedication sub-scale from the Utrecht Work Engagement 9-items Scale (UWES-9; Schaufeli et al., 2006). This scale has 5 items (e.g., I find the work that I do full of meaning and purpose), rated on a 5-point Likert scale that was adapted to provide a comparison between the current moment (during the pandemic), and the experiences before the pandemic, varying between (1) *Much less frequent than before* and (5) *Much more frequent than before*. This scale has a good reliability (ω = 0.806, 95% CI [0.79, 0.84]).

#### Role Overload

Role overload was assessed with a unidimensional scale (Thiagarajan et al., 2006; Portuguese adaptation by Matias et al., 2015), rated on a 5-point Likert scale that was adapted to provide a comparison between the current moment (during the pandemic), and the experiences before the pandemic, varying between (1) *Much less frequent than before* and (5) *Much more frequent than before*. The original scale has 6 items (e.g., I need more hours in the day to do all the things that are expected of me), but one was excluded because it only targeted parents (I seem to have more commitments to overcome than other parents I know), and another one was also not included due to a typing error in our survey platform. The final scale with 4 items has a good reliability (ω = 0.849, 95% CI [0.81, 0.88]).

#### Family-Supportive Organization Perceptions

Family-supportive organization perceptions were assessed with a 6-item scale, a shortened version developed by Booth and Matthews (2012; Portuguese translation by Santos, 2008), rated on a 5-point Likert scale that varied between (1) *Strongly disagree* and (5) *Strongly agree*. Participants were given the instruction to reply based on their organization beliefs' (and not their owns), and a sample item is “Individuals who take time off to attend to personal matters are not committed to their work.” All items were recoded to be in the positive direction. This scale has a good reliability (ω = 0.889, 95% CI = [0.86, 0.91]).

### Data Analysis

All the analyses were performed using version 27 of IBM SPSS Statistics. First, we computed the mean scores of the variables under study. To calculate the reliability of our measures, we used the McDonald's Omega (Hays and Coutts, 2020), and asked for a confidence interval of 95%. A Chi-Square independence test and a One-Way ANOVA were used to compare the sociodemographic and work variables of teachers/researchers and staff. As for the Chi-Square, comparison on the type of employment status was not performed due to the very distinctive career paths that staff and teachers/researchers have. Additionally, regarding the type of contract, we only considered comparable types of contract between staff and teachers/researchers (Open-ended/permanent, Permanent tenured, Fixed-term, Uncertain term). Normality of the distribution was assessed through the inspection of skewness and kurtosis indicators (values below |2|; Schumacker and Lomax, 2004). For the Chi-square independence test, we checked the expected frequencies and asked for the adjusted residuals to allow the interpretation of significant Chi-Square values. *T*-Tests were used to compare the two groups and Levene's homogeneity of variance test was inspected. We used a Repeated-Measures ANOVA to compare WLC before and during the pandemic for both groups and in terms of gender. For the multiple linear regression analysis, the missing values were treated using the list wise deletion and the assumptions of linearity, homoscedasticity, independence of observations and multivariate normality were checked. For all analysis, we asked for confidence intervals of 95%.

Lastly, to calculate the estimated sample size and its respective confidence level and margin of error, we used the RAOSOFT online calculator.

## Results

### Descriptive Statistics

Table 2 presents means, standard deviations and Pearson and Point-Biserial correlations of the study variables. Role overload was negatively correlated with both work dedication and family-supportive organization perceptions, and positively correlated with WLC currently and before the pandemic and time dedicated to work per day. Work dedication was only negatively correlated with current WLC. Family-supportive organization perceptions was negatively correlated with current WLC and before the pandemic. WLC before the pandemic was positively correlated with current WLC, gender, taking daily care of children and with time dedicated to work per day. Lastly, WLC before the pandemic and currently were positively correlated with taking care of children daily and with time dedicated to work per day.

**Table 2:** Descriptive statistics, and correlations [confidence intervals] between the study variables.

	**1**	**2**	**3**	**4**	**5**	**6**	**7**	**8**
1. Role Overload	–							
2. Work dedication	−0.16^*^ [−0.29, −0.03]	–						
3. Family-Supportive Organization Perceptions	−0.12 [−0.25, 0.01]	−0.02 [−0.15, 0.11]	–					
4. WLC before pandemic	0.18^**^ [0.04, 0.31]	0.09 [−0.04, 0.22]	−0.27^**^ [−0.30, −0.14]	–				
5. Current WLC	0.53^**^ [0.42, 0.62]	−0.19^**^ [−0.32, −0.06]	−0.24^**^ [−0.36, −0.10]	0.71^**^ [0.63, 0.77]	–			
6. Gender	−0.01 [−0.15, 0.13]	−0.00 [−0.14, 0.13]	−0.04 [−0.18, 0.10]	0.22^*^ [0.09, 0.35]	0.08 [−0.07, 0.22]	–		
7. Taking care of children daily	0.03 [−0.11, 0.17]	0.06 [−0.08, 0.19]	0.01[−0.13, 0.14]	0.25^**^[0.11, 0.37]	0.16^*^[0.02, 0.30]	0.06 [−0.08, 0.19]	–	
8. Time dedicated to work per day	0.21^**^ [0.08, 0.33]	0.02 [−0.11, 0.15]	−0.05 [−0.18, 0.08]	0.15^*^ [0.02, 0.27]	0.21^**^ [0.07, 0.33]	0.01 [−0.13, 0.14]	−0.10 [−0.23, 0.04]	–
*M*	3.40	3.01	3.24	2.93	3.05	–	–	8.80
*SD*	0.77	0.58	0.91	0.74	0.80	–	–	2.69

*Gender: 1 = men, 2 = women; Taking care of children daily: 0 = No, 1 = Yes*.

*Point-Biserial correlations were considered for the variables gender and taking care of children daily*.

* *p < 0.050*

** *p < 0.010*.

### WLC of Staff and Teachers/Researchers

An Independent Samples *T*-Test showed that the reported levels of current WLC differed significantly between staff (*M* = 2.86, *SD* = 0.80) and teachers/researchers (*M* = 3.23, *SD* =0.77), *t*(210) = 3.43, *p* = 0.001, with the latest reporting higher levels of WLC. There was no difference in WLC before the pandemic between both groups, *t*(231) = 1.71, *p* = 0.090, 95% CI [-0.03, 0.36].

Among staff, current WLC did not differ significantly between women and men. Also, no differences were found between women and men in the reported levels of role overload, work dedication, and family-supportive organization perceptions.

As for teachers/researchers, current WLC was higher among women (*M* = 3.37, *SD* = 0.75) than in men (*M* = 2.99, *SD* = 0.75), *t*(89) = −2.34, *p* = 0.021, 95% CI [-0.72,−0.06]. However, no differences were found between women and men in the reported levels of role overload, work dedication, and family-supportive organization perceptions.

**Figure 1:**
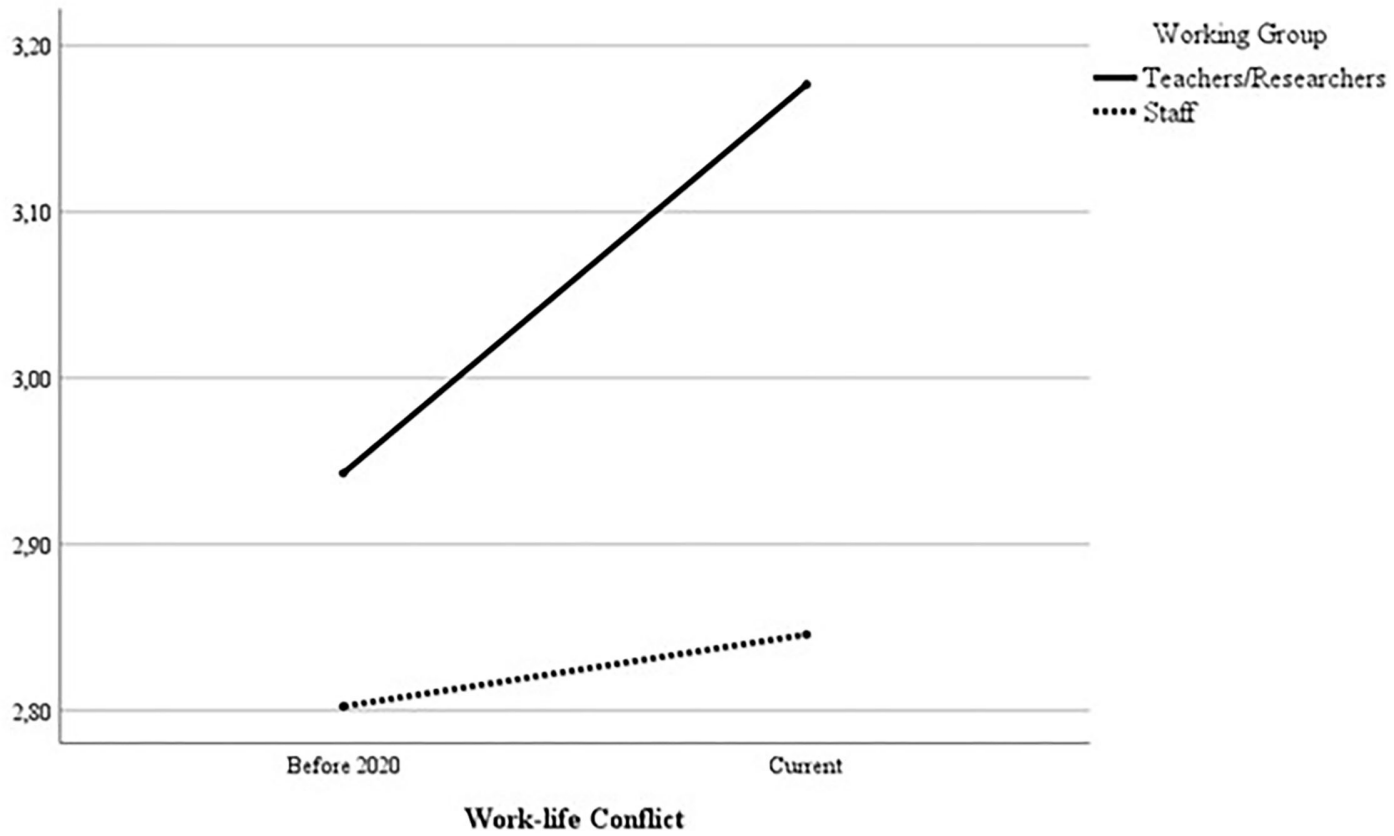
WLC before and during the pandemic among teachers/researchers and staff.

Using a Repeated Measures ANOVA, we compared the reported levels of WLC before the pandemic and currently, both among staff and teachers/researchers. There was a significant interaction between WLC and the working group (staff vs. teachers/researchers), *F*_(1, 178)_ = 4.32, *p* = 0.039. More specifically, it means that the increase of WLC was significantly more prominent among teachers/researchers (Before the pandemic: *M* = 3.01, *SD* = 0.70; Current: *M* = 3.23, *SD* = 0.77) than in staff (Before the pandemic: *M* = 2.85, *SD* = 0.71; Current: *M* = 2.84, *SD* = 0.77) (see Figure 1).

Additionally, we did not find a significant interaction between WLC, gender, and the working group, *F*_(1, 178)_ = 1.07, *p* = 0.302.

### Demands and Resources: Prediction Model of WLC

A Multiple Hierarchical Linear Regression was performed to assess the prediction of current WLC considering the role of control variables: gender, caring for children on a daily-basis, time dedicated to work per day, and WLC before the pandemic; demand variables: role overload; and resource variables: work dedication and family-supportive organization perceptions. This analysis was conducted for staff and teachers/researchers separately (see Table 3).

**Table 3:** Multiple hierarchical linear regression: predictors of current WLC for teachers/researchers and staff.

		**Teachers/Researchers**							**Staff**						
						**95% CI**		**Correlation**					**95% CI**		**Correlation**
**Model**		**B**	**β**	**t**	***p***	**LL**	**UL**	**Zero-order**	**B**	**β**	**t**	***p***	**LL**	**UL**	**Zero-order**
1	Gender	−0.03	−0.02	−0.29	0.773	−0.26	0.19	0.23	−0.09	−0.056	−0.52	0.603	−0.43	0.25	−0.01
	Taking care of children daily	0.02	0.01	0.17	0.865	−0.20	0.24	0.07	0.06	0.040	0.36	0.721	−0.29	0.42	0.18
	Time dedicated to work per day	−0.00	−0.01	−0.15	0.882	−0.04	0.04	0.27	−0.01	−0.045	−0.42	0.675	−0.07	0.05	0.13
	WLC before the pandemic	0.92	0.83	12.05	**0.000**	0.77	1.07	0.83	0.58	0.543	4.83	**0.000**	0.34	0.82	0.58
2	Gender	−0.05	−0.03	−0.58	0.566	−0.24	0.13	–	−0.10	−0.06	−0.64	0.522	−0.40	0.20	–
	Taking care of children daily	0.06	0.04	0.68	0.499	−0.12	0.24	–	−0.11	−0.07	−0.68	0.497	−0.43	0.21	–
	Time dedicated to work per day	−0.02	−0.06	−0.10	0.323	−0.05	0.02	–	−0.00	−0.01	−0.12	0.902	−0.06	0.05	–
	WLC before the pandemic	0.83	0.76	12.97	**0.000**	0.71	0.97	–	0.64	0.59	6.01	**0.000**	0.42	0.85	–
	Role overload	0.40	0.34	5.99	**0.000**	0.27	0.54	0.51	0.46	0.43	4.54	**0.000**	0.26	0.66	0.41
3	Gender	−0.05	−0.03	−0.50	0.617	−0.23	0.14	–	−0.11	−0.07	−0.79	0.431	−0.40	0.17	–
	Taking care of children	0.08	0.05	0.91	0.365	−0.10	0.26	–	−0.08	−0.05	−0.50	0.618	−0.39	0.23	–
	Time dedicated to work per day	−0.02	−0.06	−1.11	0.269	−0.05	0.02	–	−0.02	−0.07	−0.75	0.454	−0.07	0.03	–
	WLC before the pandemic	0.77	0.70	11.03	**0.000**	0.63	0.91	–	0.62	0.58	5.97	**0.000**	0.41	0.83	–
	Role overload	0.39	0.32	5.86	**0.000**	0.25	0.52	–	0.35	0.33	3.38	**0.001**	0.14	0.56	–
	Work dedication	−0.07	−0.05	−0.95	0.345	−0.22	0.08	−0.05	−0.36	−0.26	−2.69	**0.009**	−0.63	−0.09	−0.38
	Family-supportive organization perceptions	−0.11	−0.13	−2.14	**0.036**	−0.20	−0.01	−0.51	−0.04	−0.05	−0.56	0.575	−0.18	0.10	−0.19

*The bold values indicate the significant predictors of each model*.

#### Staff

##### Control Variables

The first block included gender, caring for children on a daily-basis, time dedicated to work per day and WLC before the pandemic as predictor variables. This model was statistically significant [*F*_(4, 62)_ = 6.94, *p* < 0.001, *R* = 0.56, *R*^2^ = 0.31]. Only WLC before the pandemic was a significant predictor. No significant results were found for gender, time dedicated to work per day and of caring for children. This model explains 31% of the variance of current WLC.

##### Demands

In the second block we added role overload. This model was statistically significant [*F*_(5, 61)_ = 11.43, *p* < 0.001, *R* = 0.70, *R*^2^ = 0.44] and role overload showed to be a significant predictor of current WLC.

This block added 13% to the explained variance of WLC and explains 44% of current WLC variance together with the control variables.

##### Resources

By adding work dedication and family-supportive organization perceptions, we found the model to be statistically significant [*F*_(7, 59)_ = 10.04, *p* < 0.001, *R* = 0.74, *R*^2^ = 0.49] but, along with role overload, only work dedication showed to be a significant predictor; there was not a significant effect of family-supportive organization perceptions. This block adds 5% to the explained variance and explains 49% of current WLC variance together with role overload and control variables.

The zero-order correlation aligns with these results, as WLC before the pandemic alone has a stronger correlation with current WLC, followed by role overload and work dedication.

#### Teachers/Researchers

##### Control Variables

In the first block, gender, caring for children on a daily-basis, time dedicated to work per day and WLC before the pandemic were entered as predictor variables. The results showed the model to be statistically significant [*F*_(4, 76)_ = 40.44, *p* < 0.001, *R* = 0.83, *R*^2^ = 0.68]. Only WLC before the pandemic was a significant predictor. No significant effects were found for gender, time dedicated to work per day and of caring for children. These control variables account for 68% of the variation in current WLC.

##### Demands

In the second block we added role overload. This model was statistically significant [*F*_(5, 75)_ = 54.39, *p* < 0.001, *R* = 0.89, *R*^2^ = 0.78] and role overload showed to be a significant predictor of WLC. WLC before the pandemic was still a significant predictor when role overload was added to the model.

This block added 10% to the explained variance of current WLC and, in total, explains 78%.

##### Resources

By adding work dedication and family-supportive organization perceptions, we found the model to be statistically significant [*F*_(7, 73)_ = 41.42, *p* < 0.001, *R* = 0.89, *R*^2^ = 0.80], with family-supportive organization perceptions as the only resource to be significant. Contrarily to what happened with the staff group, work dedication was not a significant predictor. This block adds 2% to the explained variance of current WLC and explains a total of 80%, together with the control variables and role overload.

The zero-order correlation aligns with these results, as WLC before the pandemic alone has a stronger correlation with current WLC, followed by role overload and family-supportive organization perceptions.

## Discussion

In this study, following the Job Demands-Resources theory (Bakker and Demerouti, 2007; Bakker and de Vries, 2021), we aimed to (i) characterize work-life conflict (WLC) among men and women workers from an HEI (staff and teachers/researchers) during the COVID-19 pandemic; and (ii) explore the major predictors of WLC for both staff and teachers/researchers, specifically job demands (role overload), and job resources (family-supportive organization perceptions and work dedication).

Teachers and researchers (and not staff) showed a significant growth in WLC between the period before the pandemic and during (H1 partially confirmed), while simultaneously showing higher levels of current WLC than staff, which answers our first research question. This result may be due to the different nature of the work tasks performed by each of these groups, which may suggest that teachers/researchers faced increased challenges associated with their tasks than staff. Specifically, their change to remote work has also implied a change to remote teaching, which poses unique constraints regarding the unavailability of teaching tools that may generate a sense of being incapable of providing students with the expected knowledge (Kulikowski et al., 2021). In turn, this may have led to a greater workload in an attempt to meet students' learning needs. Indeed, it is also important to note that teachers/researchers comprehend a group of workers where both activities—teaching and research—are most of the time cumulative. Thus, besides the increased challenges in teaching, this group also faced increased challenges to do research. As it has been noted in studies before the pandemic, quantitative demands (e.g., workload and time pressure), were already salient demands for both teachers and researchers (Naidoo-Chetty and Du Plessis, 2021).

Additionally, among teachers and researchers (and not staff), current WLC was higher for women compared to men, which is in line with most research on this topic (H2 partially confirmed). Not only women in the general population were the most affected by the pandemic, but also women academics in the HEI context. Adding to the profound changes related to work faced by teachers/researchers, women usually experience more distress from stressful life events (Kessler and McLeod, 1984), and the pandemic was not an exception. We may wonder that, for women, under the pandemic context, WLC may not have necessarily been driven from conflicting amount of tasks and responsibilities, but also to the emotional capacity to fulfill each of them. These gendered impacts of the pandemic should not be considered independently of the organization, but rather as a result of inequalities reproduced by HEIs (Pereira, 2021).

Answering the second research question, the only predictors of current WLC for staff were WLC before the pandemic (control), role overload (quantitative demand), and work dedication (personal resource). On the other hand, for teachers and researchers, both WLC before the pandemic and role overload were predictors, but work dedication seemed to be a less relevant resource, as only the perception of a family-friendly organization (organizational resource) showed a predictor effect. It appears that, for teachers/researchers, as previous research has suggested (Mishra et al., 2020; Myers et al., 2020; Górska et al., 2021), there was indeed a greater burden in terms of workload that reflects the way academics were able to manage their life roles (i.e., work-life conflict). In this line, it is worth noticing that, during the pandemic, many employees feared for the security of their employment (Kniffin et al., 2021). In the context of the HEI under study, it is interesting to note that, contrarily to staff, less than half of teachers/researchers do not have such a stable contract with the organization. According to Lin et al. (2021), under a crisis such as the COVID-19, family-friendly work practices may be helpful to alleviate such feelings of insecurity, which may explain why family-friendly organization perceptions were a resource for academics.

Regarding WLC in staff, it is not surprising that role overload was a significant predictor. Indeed, the overall situation of rapidly changing to working from home created a prone context for conflicting demands from different life domains (Sinclair et al., 2020). It is important to note, however, that this overload may have been felt at a different extent between staff and teachers/researchers depending on the responsibilities and demands associated with their roles, so we should not interpret it as having the exact same characteristics as academics, but to understand it as the result of extreme constraints that prevented the person to achieve valued goals (LePine et al., 2005). An interesting result is that work dedication, as a personal resource, was significant in preventing WLC for this group. In this HEI context, it may indicate that staff members were able to feel engaged with their work despite the pandemic hit, and therefore were more protected to a feeling of conflicting demands between work and personal life.

### Limitations

This study's results should be interpreted in the context of its limitations. Firstly, it is important to note that this sample belongs to a single HEI, limiting generalizability. It would be important to replicate this study in other HEIs to explore more contextualized conclusions and implications. Additionally, our main variable (WLC) was measured retrospectively which does not allow to completely understand the impact that demands, such as role overload, may have had in the significant increase of WLC (that was seen for teachers/researchers, but not for staff). Another important limitation, common to studies which include reporting about past experiences, is the recall bias. Although people tend to accurately recall negative experiences, positive experiences are usually overestimated (Colombo et al., 2020). Nevertheless, in this study, the risk for recall bias was minimized since the topic of the research was not highly sensitive or socially desirable, we used standardized measures to address our constructs, participants were blind to the study hypothesis, and we did not ask about excessive details of the past experience. Lastly, we should note the size of our sample because, although it seems to be representative of this specific HEI population and the margin of error is below 6%, it is still rather limited, which restricted the testing of more complex models and a deeper understanding of the study variables. The timing of the data collection (i.e., end of the semester with an intense workload due to the exams period, and approaching of summer holidays) may have been a factor for the low response rate.

### Implications

Despite of its limitations, this study offers important and new information about how workers among an HEI were able to manage their work and life roles under an unprecedent life event that rapidly changed their work settings and posed additional daily stressors. Additionally, to our knowledge, only one study (Charoensukmongkol and Phungsoonthorn, 2021) had also targeted staff members of HEI so far. Another relevant feature of this research is that we move away from the literature that solely focuses on academics' productivity and may “end up reproducing the normalization of intense and constant work” (Pereira, 2021, p. 501).

This study highlights the importance of HEIs identifying their workers characteristics and demands, and how they may specifically be linked to their role as a worker of that institution. By doing so, HEIs can easily develop and/or foster resources that are tailored to specific groups with specific needs, whether based on type of tasks (i.e., academics or staff) or gender.

As most European HEIs are currently making active efforts to promote gender-equal academic workplaces, namely by establishing Gender Equality Plans, these findings may help them design tailored and effective measures to address employees' work-life balance issues, not only considering gender, but also the different types of demands associated with each group of workers within HEI. As Corbera et al. (2020) suggest, the COVID-19 pandemic can and must be a driver for redefining academic excellence, which implies challenging the notion of scholarly productivity and fostering more family-friendly and healthy work environments.

## Data Availability Statement

The raw data supporting the conclusions of this article will be made available by the authors, without undue reservation.

## Author Contributions

MM and CG original idea of the study. MM and SM design of the study. MM coordinated the study and data collection. JF recruited participants, collected the data, and prepared data management plans. CG computed analyses and drafted a preliminary version of the article. MM, SM, and JF reviewed and edited the article. All authors have approved the final version of the manuscript for submission.

## Funding

This project has received funding from the European Union's Horizon 2020 research and innovation programme under grant agreement no: 101006560. This work was also funded by the Foundation for Science and Technology Portugal (CPUP/UIDB/00050/2020).

## Conflict of Interest

The authors declare that the research was conducted in the absence of any commercial or financial relationships that could be construed as a potential conflict of interest.

## Publisher's Note

All claims expressed in this article are solely those of the authors and do not necessarily represent those of their affiliated organizations, or those of the publisher, the editors and the reviewers. Any product that may be evaluated in this article, or claim that may be made by its manufacturer, is not guaranteed or endorsed by the publisher.
